# Post-SSRI sexual dysfunction: barriers to quantifying incidence and prevalence

**DOI:** 10.1017/S2045796024000441

**Published:** 2024-09-18

**Authors:** David Healy, Dee Mangin

**Affiliations:** 1 Data Based Medicine, Wales, UK; 2 Department of Family Medicine, McMaster University, Hamilton, ON, Canada; 3 Department of General Practice, University of Otago, Christchurch, New Zealand

**Keywords:** adverse effects, antidepressants, sexual dysfunction, suicide

## Abstract

While sexual dysfunction is a well-known side effect of taking selective serotonin reuptake inhibitors (SSRIs), in an undetermined number of patients, sexual function does not return to pre-drug baseline after stopping SSRIs. The condition is known as post-SSRI sexual dysfunction (PSSD) and is characterised most commonly by genital numbness, pleasureless or weak orgasm, loss of libido and erectile dysfunction. This article provides a commentary on the incidence and prevalence of PSSD based on a combination of academic literature as well as clinical and research experience. A number of obstacles to quantifying the occurrence of PSSD are outlined including difficulty in designing a suitable study method. Other contextual obstacles include patient embarrassment at raising sexual concerns, the response of healthcare professionals, inability to stop an antidepressant due to withdrawal issues in a proportion of patients and patient unawareness that their sexual difficulties are linked to prior medication compounded by variability of online information and a lack of information aimed at public education. A definition of PSSD with diagnostic criteria has been published. A MedDRA code for PSSD has also been introduced, but this is yet to be adopted by regulators.

## Background

Post-SSRI sexual dysfunction (PSSD) is an iatrogenic condition involving the persistence of sexual side effects after discontinuation of serotonin reuptake inhibiting antidepressants (Reisman, 2020). This group predominantly includes the selective serotonin reuptake inhibitors (SSRIs), serotonin-norepinephrine reuptake inhibitors (SNRIs), and some tricyclic antidepressants such as amitriptyline, clomipramine and imipramine. Symptoms can include genital numbness, pleasureless or weak orgasm, erectile dysfunction and loss of libido (Ben-Sheetrit *et al.*, 2015; Csoka *et al.*, 2008; Healy *et al.*, 2018; Hogan *et al.*, 2014; Kauffman and Murdock, 2007; Muquebil Ali Al Shaban Rodríguez *et al.*, 2017; Patacchini and Cosci, 2020; Waldinger *et al.*, 2015; Waraich *et al.*, 2020). It affects all ages, both sexes and all ethnic groups (Patacchini and Cosci, 2021; Stinson, 2013).

The UK’s Medicines and Healthcare products Regulatory Agency (MHRA) have indicated that their first report of a sexual dysfunction continuing after an SSRI was stopped involved fluoxetine in 1991 (MHRA, 2019). PSSD was first reported in the medical literature in 2006 (Bahrick, 2006; Csoka and Shipko, 2006). In 2011, the US product information for fluoxetine was amended to include a warning about the possible persistence of sexual side effects after discontinuation (Eli Lilly and Company, 2011). Beginning in 2012, the Netherlands Pharmacovigilance Centre Lareb have published several reports about the condition (Chinchilla Alfaro *et al.*, 2022; Ekhart and van Puijenbroek, 2014; Lareb, 2012). In 2013, the fifth edition of the Diagnostic and Statistical Manual of Mental Disorders stated: ‘In some cases, serotonin reuptake inhibitor-induced sexual dysfunction may persist after the agent is discontinued’ (American Psychiatric Association, 2013, p. 449). In response to a petition (Healy, 2018) and supporting data supplied by the medication safety group RxISK.org (Healy *et al.*, 2019), the European Medicines Agency and Health Canada recommended changes to SSRI and SNRI product labels to include information about persistent sexual dysfunction after stopping the medication (European Medicines Agency, 2019; Health Canada, 2021). Diagnostic criteria for PSSD were published in 2022 (Healy *et al.*, 2022). In 2024, Australia’s Therapeutic Goods Administration issued a safety update advising that all SSRIs and SNRIs would carry warnings about persistent sexual dysfunction after drug cessation (Therapeutic Goods Administration, 2024). In the literature, possible mechanisms have been proposed and there has been discussion of attempts to remedy the condition, but this has yet to lead to any established treatments (Bala *et al.*, 2018; Calabrò *et al.*, 2019; Giatti *et al.*, 2024; Gül *et al.*, 2024; Healy *et al.*, 2020; Klaas *et al.*, 2023; Reisman, 2020; Reisman *et al.*, 2022; Waldinger *et al.*, 2015).

## Studies

A gap in our knowledge of PSSD is understanding the prevalence of this condition and the incidence in people starting on SSRIs as well as a detailed description of the epidemiology, natural history and prognosis. Despite calls for post-marketing studies (Farnsworth and Dinsmore, 2009; Kauffman, 2008), epidemiological research remains scarce owing to the difficulty in designing a suitable method. For example, it would be unethical to run a randomized controlled trial that carried a risk of participants developing the condition as its outcome. Suitable validated tools to measure outcomes based on diagnostic criteria are also needed. It has been noted that some validated questionnaires such as the Arizona Sexual Experience Scale and the Changes in Sexual Functioning Questionnaire do not include questions about genital sensation and may not be suitable to capture the nuances of PSSD (Bahrick, 2008; Raj, 2020).

Pharmaceutical company studies do not often include follow-up to check for enduring side effects. However, a small number of studies in the PSSD literature do have relevant data. One clinical study reported that amineptine, a tricyclic antidepressant with no action on the serotonin system, did not cause sexual dysfunction in patients treated solely with this drug. However, when a group of patients with SSRI-induced sexual dysfunction were switched from an SSRI to amineptine, 55% still had sexual dysfunction after 6 months (Montejo *et al.*, 1999). A large placebo-controlled study into the use of sertraline as a treatment for premature ejaculation found that the ejaculation-delaying effect of the drug persisted for 34% of participants 6 months after it was discontinued (Arafa and Shamloul, 2006). A healthy volunteer study that assessed the effects of paroxetine on sperm and sexual function reported that brief sexual function inventory (BSFI) scores for erectile and ejaculatory functions had not returned to baseline four weeks after drug discontinuation, with 9% of patients complaining of more than mild dysfunction (Tanrikut *et al.*, 2010). The BSFI questionnaire does not include questions about genital sensation or orgasm intensity.

In some unpublished phase 1 trials, over 50% of healthy volunteers had severe sexual dysfunction that in some cases persisted after treatment stopped (SmithKline Beecham, 2013). However, these data are not publicly available.

A recent study by Lüning in 2019 appears to be the first reported attempt to investigate the prevalence of PSSD (Lüning, 2019). Former antidepressant users were surveyed for evidence of persisting sexual effects. Recruitment methods included online advertisements and the distribution of flyers in various locations. In addition to using validated questionnaires, further questions were included to look for symptoms that were specific to antidepressants. From a sample size of 76 participants, the study reported that 52.6% (*n* = 40) of participants suffered from persisting sexual dysfunction, while 26.3% (*n* = 20) of participants suffered from genital anaesthesia and/or nipple insensitivity, the latter figure being suggestive of PSSD. A number of limitations to this study were also discussed including possible selection bias, possible confounding from current medications, and lack of questions about sexual baseline. The author noted that while the results should be interpreted with caution, they ‘provide a first indication of the prevalence of these problems in a sample of people who previously used antidepressant medication’.

Ben-Sheetrit et al. carried out a 19-year retrospective cohort analysis by searching patient records in a computerised database for prescriptions of phosphodiesterase 5 (PDE5) inhibitors for erectile dysfunction after the discontinuation of serotonergic antidepressants (Ben-Sheetrit *et al.*, 2023). The study reported a 0.46% risk of developing an irreversible sexual dysfunction. Limitations included exclusion of females, a focus on erectile dysfunction rather than other forms of sexual dysfunction, the exclusion of comorbidities, and a reliance on patients seeking medical help. The authors acknowledged that these and other limitations mean the study may have underestimated the true prevalence of the condition. In addition, it is important to note that PDE5 inhibitors are not a treatment for PSSD and have no direct effect on the loss of sensation. Their use for the condition may be relatively uncommon as one survey reported that only 23% (26/115) of male subjects had used tadalafil or similar (Patacchini and Cosci, 2021).

The widely varied results of these studies may reflect the very different methodologies and the inherent biases discussed, thereby highlighting the importance of considering the results of any study within its proper context and the need for better information.

## Healthcare professionals

Antidepressants are often prescribed without any discussion of implications for sexual function. A study of 239 previous antidepressant users found that only 12% reported being counselled regarding potential sexual side effects while taking an antidepressant (Studt *et al.*, 2021). Similarly, doctors do not routinely enquire about the resolution of sexual side effects after stopping an antidepressant and are therefore unlikely to discover a case of PSSD unless a patient makes a link themselves, and further, feels able to bring it to their attention.

It is not uncommon for patients to have difficult experiences with healthcare professionals when trying to seek help for symptoms they suspect are PSSD. Reports of unhelpful, dismissive and hostile responses have been documented in the medical literature (Healy *et al.*, 2019). These include patients having their suspicions ridiculed, being advised to find a different sexual partner, or having their symptoms attributed to some kind of ongoing mental health condition. Some patients have taken academic literature to consultations only to have it ignored. In a recent media article, one patient described being sectioned and placed involuntarily into psychiatric care as a result of seeking help for PSSD from a local mental health service (The Guardian, 2024). This type of behaviour from healthcare professionals creates two problems in terms of limiting visibility of the condition and in turn an understanding of the epidemiology. First, these cases are not being properly recorded in the medical notes in a way that would provide searchable data for research purposes because a link to medication is not ordinarily recognised. Second, patients often stop consulting healthcare professionals because of the attitudes they encounter, either changing clinicians or never raising the issue again, and so these cases disappear from clinical practice and records. Patients who have not yet spoken to their doctor, but have read about the kind of response they will probably receive, may decide against it. The lack of available treatment also means that patients may feel less motivated to seek out medical advice.

Invalidating responses revolve around the notion that the medication is no longer in the body and therefore any enduring problem must be psychological. There are many recorded examples of drug-induced permanent disability with the most common drug classes including central-nervous-system agents, antimicrobials, vaccines and antineoplastics (Kelly, 2001). A very relevant precedent is tardive dyskinesia, a potentially permanent movement disorder that can occur after stopping antipsychotics. Tardive dyskinesia has been well-recognised for decades and has similarities to PSSD in that it can appear on medication and remain after stopping, or sometimes worsens when the medication is stopped. Additionally, no mental health disorder causes anaesthesia of the genitals, whereas it is a documented effect of serotonin reuptake inhibiting antidepressants (Colpi *et al.*, 1991; Deisenhammer and Trawöger, 1999; Ellison and DeLuca, 1998; Measom, 1992; Michael and Andrews, 2002; Michael and Mayer, 2000; Neill, 1991; King and Horowitz, 1993; Yilmaz *et al.*, 1999). PSSD sufferers also typically have normal sexual functioning prior to taking an antidepressant (Patacchini and Cosci, 2021).

## Patients

The expression of PSSD can vary in severity between individuals, and some people may misattribute symptoms, particularly if they experienced an improvement in sexual function upon stopping the antidepressant. For example, some patients regain the ability to achieve orgasm after previously being incapable of orgasm while on the antidepressant, but it will often feel weaker than before. As they are no longer on the drug, people can attribute changes like this to ageing for instance, especially as a large proportion of patients remain on SSRIs for many years after initiation (Mangin *et al.*, 2018).

Animal studies have reported sexual deficits in rodents exposed to antidepressants prenatally and during early life (Simonsen *et al.*, 2016). If this is paralleled in humans as suggested by one study (Lorenz, 2020), those affected do not have a sexual baseline to compare to, control comparison is awkward, and it is unlikely they would link their problem to the temporally distant antidepressant exposure which may be indirect.

Males sometimes refer to their condition as an erectile dysfunction, while females commonly frame it as a loss of libido. Details of changes in genital sensation and orgasm intensity are not always volunteered unless asked about directly. This may be out of embarrassment, because the questions asked are not specific enough, or because they did not realise it was important. These cases may fall through the cracks if looking for patients who use specific terms such as genital numbness and pleasureless orgasm. There can also be confusion about genital numbness as patients may describe a reduction in either tactile or erotic sensation, or a combination of both. Quantitative sensory testing of the genitals may be helpful in elucidating tactile insensitivity (Waraich *et al.*, 2020) but is not widely available. Erotic sensation cannot be easily assessed and is a subjective experience.

Embarrassment can deter patients from speaking to their doctor about sexual concerns. If a patient attempts to raise the issue but their doctor appears uncomfortable and does not make the patient feel at ease, they may decide not to mention it further. The sensitive nature of PSSD also means that very few people are willing to speak to peers or publicly about having the condition.

A systematic review of the literature on antidepressant withdrawal found that 56% of people who attempt to come off antidepressants experience withdrawal effects, with 46% of those describing them as severe (Davies and Read, 2019). Being unable to stop an antidepressant due to withdrawal symptoms, or having to reinstate it within a short period, also potentially masks the presence of any enduring post-treatment problems such as PSSD.

In the absence of robust information on the epidemiology of PSSD and potential treatment or prevention, the quality of online information is variable and there is scope for misunderstanding. The condition is sometimes incorrectly portrayed as a catch-all diagnosis for any post-antidepressant effects. A need to believe that recovery is possible and a need to find success stories sometimes leads sufferers to employ dubious interpretations of the word ‘recovery’. For example, some sufferers claim to have recovered because they experienced a degree of improvement despite having ongoing problems. The number of people involved in online groups may not give an accurate indication of the scale of the problem as some people read forums without joining or participating, while some others follow them for a while but become jaded due to lack of progress and decide to move on. There can also be biases in any online group driven by the forum rules and the opinions of the moderators.

As discussed, the manifestations of PSSD vary between individuals and the same is true of the wider impact on their lives. PSSD can lead to marriage break-up and job loss (Healy *et al.*, 2018). One of the authors (DH) is aware of at least 20 cases of people who have committed suicide as a result of the condition. Conversely, the loss of libido may mean that some sufferers are no longer interested in sexual activity and may be relatively unconcerned about having the condition.

## Conclusion

It is not known how many patients, if any, fully regain their original genital sensation, orgasm intensity, and other domains of sexual functioning after using a serotonin reuptake inhibiting antidepressant. This has never been properly investigated and described, and there is an urgent need to understand the incidence, prevalence and natural history of PSSD, with a focus on informing prevention as well as investigating treatments.

The combination of clinical features and the course of PSSD defeat big data approaches as outlined. There might be some hope if PSSD was a longstanding condition, known about for decades and recognised as such, so that databases could be searched for coding terms or textwords. But it has only been recognised in drug labels since 2019, and has only since then been given a Medical Dictionary for Regulatory Activities (MedDRA) code which at present does not appear to have been adopted by regulators. In an online comment, one of the early authors of the PSSD literature noted a gap in the ability of regulators to recognise enduring medication issues:

*I sent in several case reports of persistent sexual dysfunction to the FDA in the late 90’s however they were recorded as problems during medication and not afterward. It turns out that anything after stopping the drug isn’t considered a side effect. (Shipko, 2017*)

There is a general lack of awareness and understanding of the condition among both patients and healthcare professionals. Against this backdrop of underrecognition, investigating the number of cases presenting at urology clinics for example, or surveying clinicians about how often they encounter the condition, would also be unlikely to produce meaningful results.

There has been relatively little information aimed at educating the general public about PSSD that would support recognition by patients. From 2010, when attempting to raise the issue with US and UK media, one of the authors (DH) was repeatedly told that we in the media do not want to deter people from taking their antidepressants.

While many cases of PSSD could be relatively straightforward to diagnose in clinical practice where full details of medical history and clinical presentation are available, there are several factors that obscure the visibility of PSSD across a wider population. This presents a significant challenge to epidemiologists and an information gap of substantial importance to patients and clinicians.
